# Small serine recombination systems ParA‐*MRS* and CinH‐*RS2* perform precise excision of plastid DNA

**DOI:** 10.1111/pbi.12740

**Published:** 2017-05-16

**Authors:** Min Shao, Ann Blechl, James G. Thomson

**Affiliations:** ^1^ UC Davis Department of Plant Sciences Davis CA USA; ^2^ USDA‐WRRC‐ARS Crop Improvement and Genetics Research Unit Albany CA USA

**Keywords:** ParA‐MRS, CinH‐RS2, site‐specific recombination, marker excision

## Abstract

Selectable marker genes (SMGs) are necessary for selection of transgenic plants. However, once stable transformants have been identified, the marker gene is no longer needed. In this study, we demonstrate the use of the small serine recombination systems, ParA‐*MRS* and CinH‐*RS2,* to precisely excise a marker gene from the plastid genome of tobacco. Transplastomic plants transformed with the pTCH‐*MRS* and pTCH‐*RS2* vectors, containing the visual reporter gene *DsRed* flanked by directly oriented *MRS* and *RS2* recognition sites, respectively, were crossed with nuclear‐genome transformed tobacco plants expressing plastid‐targeted ParA and CinH recombinases, respectively. One hundred per cent of both types of F_1_ hybrids exhibited excision of the *DsRed* marker gene. PCR and Southern blot analyses of DNA from F_2_ plants showed that approximately 30% (CinH‐*RS2*) or 40% (ParA‐*MRS*) had lost the recombinase genes by segregation. The postexcision transformed plastid genomes were stable and the excision events heritable. The ParA‐*MRS* and CinH‐*RS2* recombination systems will be useful tools for site‐specific manipulation of the plastid genome and for generating marker‐free plants, an essential step for reuse of SMG and for addressing concerns about the presence of antibiotic resistance genes in transgenic plants.

## Background

Plastids of higher plants are cellular organelles with small highly polyploid genomes that contain their own transcription‐translation machinery. The concept of plastid genetic engineering was developed in the 1980s (Daniell and McFadden, 1987). Plastid transformation generally results from homologous recombination of transgenic DNA that replaces the corresponding chloroplast DNA. Plants with transformed plastid genomes are termed transplastomic (Maliga, 1993). Although plastid transformation has been achieved by the biolistic particle approach (Svab *et al*., 1990) or the polyethylene glycol mediated method (PEG), the former has become the favoured method of plastid transformation because of its simplicity and higher transformation efficiencies (Ahmad *et al*., 2010). Plastid transformation offers several advantages over nuclear transformation, including targeted insertion of the transgenes at a determined position via homologous recombination, high levels of transgene expression, multigene expression in a single transformation event and maternal inheritance of plastid genes to prevent outcrossing of transgenes to wild relatives via pollen (Daniell *et al*., 2016). Because of these advantages, the plastid genome DNA (ptDNA) of higher plants is an attractive target for biotechnology applications. Positive plastid gene transformation has been carried out in tobacco (Svab *et al*., 1990), Arabidopsis (Sikdar *et al*., 1998), potato (Sidorov *et al*., 1999), tomato (Ruf *et al*., 2001), carrot (Kumar *et al*., 2004) and rice (Khan and Maliga (1999). However, plastid transformation is routine only in tobacco as its transformation efficiency is much higher than in other plants (Maliga, 2004). Thus, most experiments have been described in tobacco, which has become the model species for plastid transformation. To date, the transcriptionally active intergenic region between the *trnI‐trnA* genes, within the rrn operon, has become the most commonly used site of integration and is located in the IR regions of the chloroplast genome. Expression level of transgenes from this location is among the highest reported (De Cosa *et al*., 2001). For addition information on plants transformed, regions targeted, foreign proteins produced and levels of expression reference, see Daniell *et al*. (2016).

Selectable marker genes (SMGs) are necessary tools in plastid transformation. Most SMGs confer antibiotic‐ or herbicide resistance traits, and often reside in the final product of genetically modified (GM) plants. However, SMGs are unnecessary once a transplastomic plant has been obtained. The presence of these resistance traits in engineered plants, and consequently in food, feed and the environment, are of specific focus to government regulation in many countries. Moreover, high levels of SMGs expression in transplastomic plants might result in a metabolic burden for the host plants. Additionally, there are a number of desirable traits and genes of interest for incorporation into plants, but a limited number of SMGs available for practical use. For plastid transformation selection, spectinomycin (*aadA*) is predominantly used, although kanamycin (*nptII* or *aphA‐6*) and chloramphenicol (CAT) genes have been successfully shown functional for primary transformation (Chong‐Pérez and Angenon, 2013; Li *et al*., 2011). Very recently a publication describing the bifunctional resistance gene *aac(6)‐Ie*/*aph(2)‐Ia* in combination with the antibiotic tobramycin also appears to be an effective alternative for plastid manipulation (Tabatabaei *et al*., 2017). For addition information on SMG's, conditions of use, plants transformed, optimization and techniques reference, see Bock (2015). However, their initial use prevents the reuse of the same SMG when a second round of transformation is required for transplastomic plants. Further, with the development of cost‐effective edible vaccines becoming a reality (Su *et al*., 2015), the requirement of marker gene removal for public acceptance and commercialization is critical. Therefore, the development of strategies to produce SMG‐free transplastomic plants is an important objective in plant plastid biotechnology research.

While the first published strategy to remove the selectable marker utilized homologous recombination (Iamtham and Day, 2000), the use of specific recombinases has become a viable alternative to produce marker‐free transplastomic plants. Cre/lox was the first site‐specific recombination system utilized to excise the SMG from the plastid genome (Corneille *et al*., 2001; Hajdukiewicz *et al*., 2001). Cre activity was introduced by nuclear transformation and marker‐free transplastomic plants were obtained in tissue culture. Cre was then segregated away in the progeny**.** Unfortunately, Cre‐mediated marker excision can also facilitate the undesirable deletion of ptDNA sequences via the recognition of repeated nonlox sequences resulting in mutations of target plant plastid genome (Corneille *et al*., 2001; Hajdukiewicz *et al*., 2001; Tungsuchat *et al*., 2006). The large serine subfamily recombinases phiC31 and Bxb1 were also shown to excise the SMGs (Kittiwongwattana *et al*., 2007; Shao *et al*., 2014). The absence of pseudo‐att sites in plastid genomes makes phiC31 and Bxb1 preferred alternatives to Cre for plastid marker excision (Lutz and Maliga, 2007; Shao *et al*., 2014).

The site‐specific recombinase family can be divided into two basic groups: the tyrosine and serine recombinases. This split is based on the active amino acid (Tyr or Ser) within the catalytic domain of the enzymes in each subdivision. The serine recombinase subdivision has two distinctive members with the division being based on size of the enzyme. PhiC31 and Bxb1 belong to the large serine subfamily and ParA‐*MRS* and CinH‐*RS2* site‐specific recombination systems belonging to the small serine (Wang *et al*., 2011). While recombination mediated by the small serine recombinases requires identical recognition sites, only intramolecular excision events are detected. Research has determined that owing to conformational strain, small serine recombinases cannot adopt a stable synaptonemal complex that will enable inter‐molecular recombination (i.e. integration) (Mouw *et al*., 2008). Therefore, excision‐mediated DNA removal by the small serine recombinases is considered unidirectional. The ParA‐*MRS* system originated from the plasmid operon parCBA and is responsible for the maintenance of broad host range plasmids RK2 and RP4. The 222 aa ParA recombinase recognizes a 106‐bp recombination site *MRS* (multimer resolution site) (Gerlitz *et al*., 1990; Moon *et al*., 2011; Thomson and Ow, 2006). The CinH‐*RS2* system was discovered in the *Acetinetobacter* plasmids pKLH2, pKLH204 and pKLH205, where the 189 aa CinH recombinase recognizes a 119‐bp recombination site *RS2* (Kholodii, 2001; Moon *et al*., 2011; Thomson and Ow, 2006). These excision‐only systems have identical recombination sites, generally known as resolution (*res*) sites. In principle, the excision‐only systems should be effective alternatives to bidirectional systems for use in DNA editing (Thomson *et al*., 2009). ParA‐*MRS* and CinH‐*RS2* are considered unidirectional recombination systems that differ from the bidirectional systems Cre/lox, FLP/FRT and R/RS, in that the relative placement of the participating recombination sites and their orientation to one another may lead to deletion, inversion, translocation or integration (Thomson *et al*., 2009). Unidirectional recombinase system ParA‐*MRS* or CinH‐*RS2* can only recombine *MRS* or *RS2* sites, respectively, when both participating substrates are in the same orientation on the same molecule. However, as they do not catalyze intermolecular reactions, the excision reaction is not considered reversible, and in a sense analogous to other recombination systems such as phiC31 and Bxb1 (Thomason *et al*., 2001; Wang *et al*., 2011). Additionally, ParA‐*MRS* and CinH‐*RS2* systems have longer recognition sequences than the Cre‐lox, which reduces the probability of unintended recombination with native (cryptic) host sequences (Thomson and Ow, 2006).

ParA‐*MRS* and CinH‐*RS2* recombinases have been shown to efficiently excise plasmid DNA in transformed fission yeast *Schizosaccharomyces pombe* (Thomson and Ow, 2006). ParA and CinH recombinases were also effective in catalyzing nuclear DNA excision in transgenic *Arabidopsis thaliana* or tobacco plants (Moon *et al*., 2011; Thomson *et al*., 2009), but these two systems have yet to be characterized in the plant plastid. Here, we report the precise excision of plastid DNA by the small serine recombinases ParA‐*MRS* and CinH‐*RS2*.

## Results and discussion

### Experimental design

**Figure 1:**
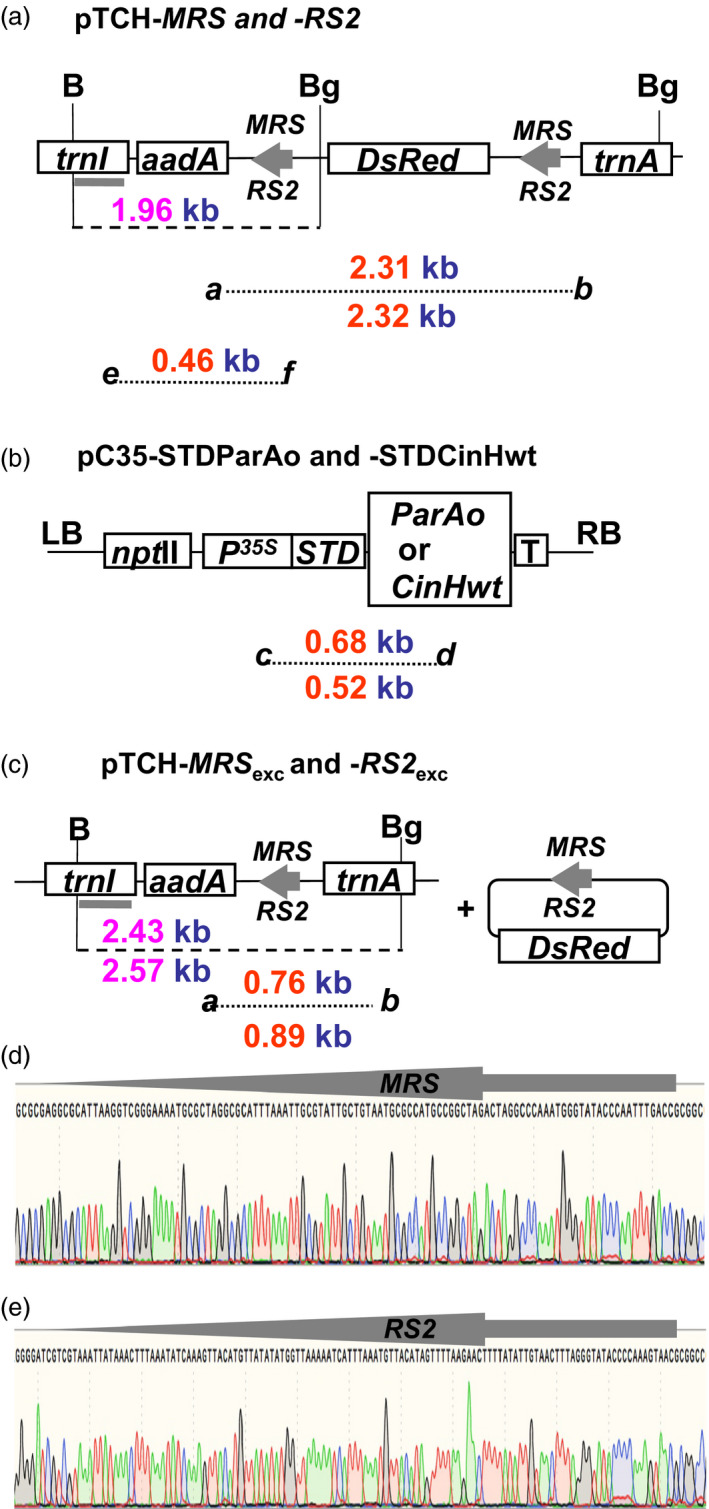
Vectors and sequences used to demonstrate precise marker gene excision from plastid DNA by ParA‐*MRS* and CinH‐*RS2* systems. (a). The pTCH‐*MRS* or pTCH‐*RS2* constructs contain either two *MRS* (for ParA excision) or *RS2* (for CinH excision) sites (grey arrows), respectively, flanking the *DsRed* gene driven by the *psbA* promoter. The locations of *Bgl* II (Bg) and *Bam*H1 (B) sites used in genomic Southern analyses are shown along with the predicted site of the fragment that hybridizes to the TRN probe (grey bar below *trnI* region). The sizes of the amplicons generated for each construct by primer pairs ***a–b*** and ***e–f*** (Table 1) are shown below the diagram. *TrnI*—chloroplast gene encoding isoleucine tRNA; *trnA*—chloroplast gene encoding alanine tRNA; *aadA*—spectinomycin 3”‐adenyltransferase gene; *DsRed*—red fluorescent protein gene; *MRS*—ParA recombinase recognition sequence; *RS2*—CinH recombinase recognition sequence; *P* ^*35S*^—Cauliflower Mosaic Virus 35S promoter. (b). p35SSTDParAo or p35SSTDCinHwt contain the recombinase expression cassettes. The size of the amplicons generated for each construct by primer pair ***c–d*** (Table 1) is shown below the diagram. *ParAo*—plant codon optimized ParA coding sequence; *CinHwt*—CinH wild‐type coding sequence; *npt* II—neomycin phosphotransferase II gene; *STD*—tobacco Rubisco stroma‐targeting domain; LB and RB—left and right, respectively, borders of T‐DNA; T–Nos 3’ terminator. (c). Excision products of site‐specific recombinase removal the *DsRed* gene from the plastid genome. Panels (d) and (e); Sequence of the PCR products containing ParA (*MRS*) and CinH (*RS2*) recognition sites after excision—arrow between *aadA* and *trnA* genes in panel **c**.

The experimental strategy used in this study is illustrated in Figure 1a–c. Two separate sets of constructs were designed as follows. The pTCH‐*MRS* and pTCH*‐RS2* plasmids (Figure 1a) were designed for detection of site‐specific excision in chloroplasts. They contain *DsRed* (the optimized *Discosoma* red fluorescent protein coding sequence, Bevis and Glick, 2002) flanked by the recognition sites *MRS* or *RS2* in direct orientation for excision by recombinases ParA or CinH, respectively. They also contain *trn* sequences to facilitate homologous recombination with the *trn* sequences in the tobacco plastid genome and the spectinomycin marker gene *aadA* coding sequence for selection after biolistic transformation of tobacco SR1. The second set of plasmids is designed for nuclear transformation of recombinases (Figure 1b). They contain ParA or CinH recombinase coding sequences *ParAo* or *CinH,* fused to the STD plastid targeting sequence, all driven by the 35S promoter. The pC35‐STDParAo and pC35‐CinHwt plasmids also contain the kanamycin selectable marker gene *npt*II for selection of nuclear DNA SR1 transformants after *Agrobacterium*‐mediated transformation. Crosses of pTCH‐*MRS* or pTCH‐*RS2* transplastomic female plants with pollen from the corresponding transgenic pC35‐STDParAo and pC35‐CinHwt plants brings together the site‐specific recombinase with its directly oriented recognition sequences in the plastid genome. The expected site‐specific deletion yields the structures diagrammed in Figure 1c. A single recognition site remains in the plastid genome, and the DsRed gene is lost. The change can be detected by PCR with primer pair ***a–b*** (Figure 1a and c and Table 1). The recombinase nuclear transgene can be segregated away in subsequent generations, leaving the transplastomic plants that lack the DsRed marker gene.

**Table 1:** Primers for DNA analysis

Detection purpose	Primer names^a^	Primer sequence (5′ to 3′)	PCR conditions (Anneal Temp/Extension time/cycles)
ParA excision	***a*** Spec 300 F65 ***b*** CH7300 R65	CCAGCTAAGCGCGAACTGCAATTTGGAGAATGG CCTCCTATAGACTAGGCCAGGATCGCTCTAGATGC	63 C 72C 45 Sec 26×
ParAo nuclear transformation	***c*** ParABHI F58 ***d*** ParAomSpeI R60	AGTCGGATCCATGGCGACCAGGGAGC AGTCACTAGTCTAAGCCCTCTTGTTTTGCT	58 C 72 C 45 Sec 32×
CinHwt excision	***a*** Spec 300 F65 ***b*** CH7300 R65	CCAGCTAAGCGCGAACTGCAATTTGGAGAATGG CCTCCTATAGACTAGGCCAGGATCGCTCTAGATGC	63 C 72C 45 Sec 26×
CinHwt transformation	***c*** CinHwt 10 F62 ***d*** CinHwt 500 R58	CCAAAAAGTAGGGTATGTGCGAGTGAGTTCG CCTGTCTACTGATACCAAACGCTTTTGCC	58 C 72C 45 Sec 35×
Chloroplast transformation	***e*** Spec300 F65 ***f*** Spec700 R66	CCAGCTAAGCGCGAACTGCAATTTGGAGAATGG CGCCTTTCACGTAGTGGACAAATTCTTCCAACTGATCTGCG	60 C 72 C 45 Sec 30×
Southern blot probe	TRN1.2 F63 TRN1.2 R63	CCACCACGGCTCCTCTCTTCTCG GCCATCCTGGACTTGAACCAGAGACCTCGCCCGTG	58 C 72 C 30 Sec 34×
DSRed gene^b^	DSRed 190 F61 DSRed 540 R62	CCAGTTCCAGTACGGCTCCAAGG GTAGATGGACTTGAACTCCACCAGGTAGTG	58 C 72 C 30 Sec 32x

a Letters at beginning of names refer to labels in Figure 1a–c.

b See Supplemental Fig S1 for details.

### Plastid target plants

**Figure 2:**
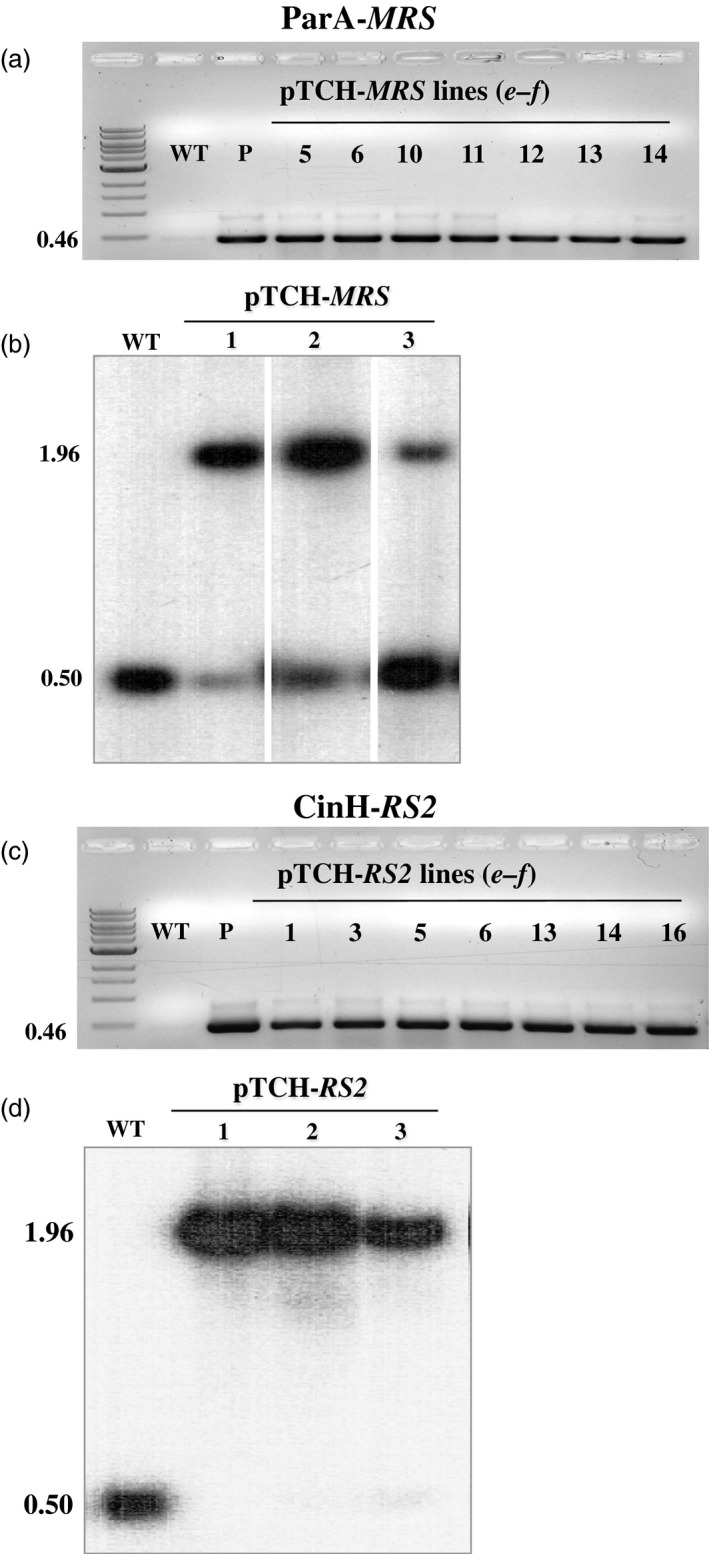
Molecular analysis transplastomic plant DNA. (a) & (c). PCR analysis of spectinomycin‐resistant regenerants. DNAs were amplified using the ***e‐f*** primer pair (Table 1, Figure 1a) with the expected size of 0.46 kb shown to the right. Panel a. DNAs from WT: untransformed negative control P: positive control (pTCH‐*MRS* vector); Lanes 5, 6, 10,11, 12, 13, 14, pTCH‐*MRS* transformants; Panel **c**. DNAs from WT; P: positive control (pTCH‐*RS2* vector); Lanes 1, 3, 5, 6, 13, 14 and 16 from, pTCH‐*RS2* transformants. M, DNA ladder. (b) & (d). Southern blot analysis of PCR‐positive transplastomic plants with probe TRN (grey bar below diagram in Figure 1a). DNAs from untransformed (WT) and transplastomic plants (Lanes 1‐3) were digested with *Bam* HI and *Bgl* II. The sizes of the hybridizing fragments are shown to the left.

Transformation of SR1 tobacco plastids was carried out using the biolistics protocol previously described (Verma *et al*., 2008). Three bombardments each were performed with the vectors pTCH‐*MRS* and pTCH‐*RS2*. After the first round of spectinomycin selection, 12 (Figure 2a) and 16 (Figure 2c) shoots, respectively, contained the vector pTCH‐*MRS* and pTCH‐*RS2* DNA, as assessed by PCR using primer pair ***a–b*** (Figure 1a, Table 1). All PCR positive transplastomic shoots were cut and placed on RMOP media containing 500 mg/L spectinomycin for a second round of selection. Again, the presence of the pTCH‐*MRS* and pTCH ‐*RS2* vector DNA was verified by PCR and sequencing of the amplicons. DNAs from three pTCH‐*MRS* transplastomic lines (TCH‐MRS1, TCH‐MRS2 and TCH‐MRS3) and three pTCH‐*RS2* transplastomic lines (TCH‐RS1, TCH‐RS2 and TCH‐RS3) were further analysed by Southern blots analysis for precise DNA integration into the plastid genome (Figure 2b, d). Hybridization with a TRN probe to Bam*HI*‐Bgl*II*‐cleaved DNA revealed the 1.96‐kb band expected from the cleaved TCH‐*MRS* and TCH‐*RS2* DNAs (Figure 1a), and a second 0.50‐kb band expected from the wild‐type plastid copy of *trnI*. Southern blot positive lines were used to test excision function by crossing with the recombinase expression lines. Results from the Southern blot indicated that the TCH‐*RS2* lines were homoplastomic while the TCH‐*MRS* still appeared heteroplastomic after two rounds of spectinomycin selection. However, the lines were felt sufficient to proceed with a proof of concept study as molecular characterization for recombinase‐mediated excision events would discern wild type from transformed from recombinase excised.

### Recombinase expression plants

**Figure 3:**
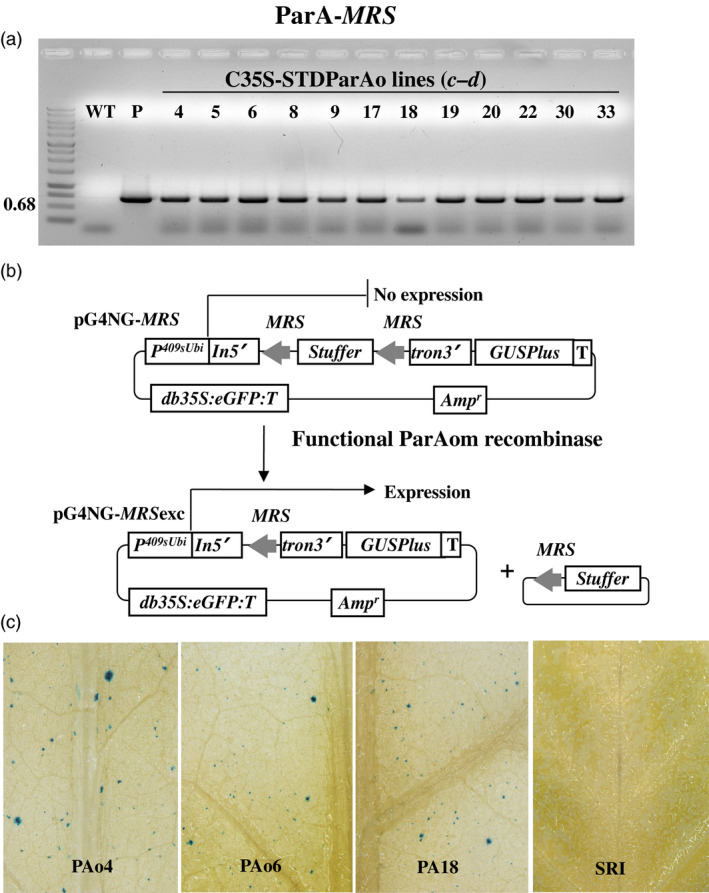
ParA transgenic plants and expression activity detection. (a). PCR analysis of kanamycin‐resistant regenerants. DNA from kanamycin‐resistant shoots was amplified by primer pair ***c–d*** (Figure 1b, Table 1) to give a 0.68 fragment that includes part of the 35S promoter, stroma targeting domain and part of the *ParAo* coding region. Amplified DNA products are from WT—untransformed tobacco negative control, P—C35SSTDParAo plasmid DNA as positive controls, and kanamycin resistant shoots (numbered lanes). (b). Schematic representative of constructions and strategy to detect recombinase activity in a transient assay. In vector pG4NG‐*MRS* GUSPlus reporter gene expression is inhibited due to the presence of a terminator‐rich ‘stuffer’ sequence in the intron (In5'tron3’). This stuffer will be excised by site‐specific recombination in cells containing ParA. The resultant pG4NG‐*MRS* exc plasmid express the GUSPlus reporter. *p409sUbi*—potato ubiquitin promoter; *T*—3'Nos terminator; *In5’*—maize ubiquitin first intron 5’ fragment; *tron3’*—maize ubiquitin first intron 3’ fragment; *GUSPlus*—β‐glucuronidase gene sequence; *db35S*‐double enhanced CaMV 35S promoter; *eGFP*‐enhanced Green Fluorescent Protein gene; *MRS*‐ParA recombinase recognition sequences; Amp—Ampicillin resistance gene. (c). Detection of site‐specific recombinase activity with the pG4NG‐*MRS* vector. Leaves from C35SSTDParAo transgenic plants after bombardment with the pG4NG‐*MRS* plasmid, histochemical staining to detect β‐glucuronidase activity, (blue spots) indicating recombinase‐mediated excision and gene activation. Leaves were from WT, nontransformed SR1 tobacco, the negative control, three *ParAo* expression transgenic plants (PAo4, PAo6 and PAo18).

**Figure 4:**
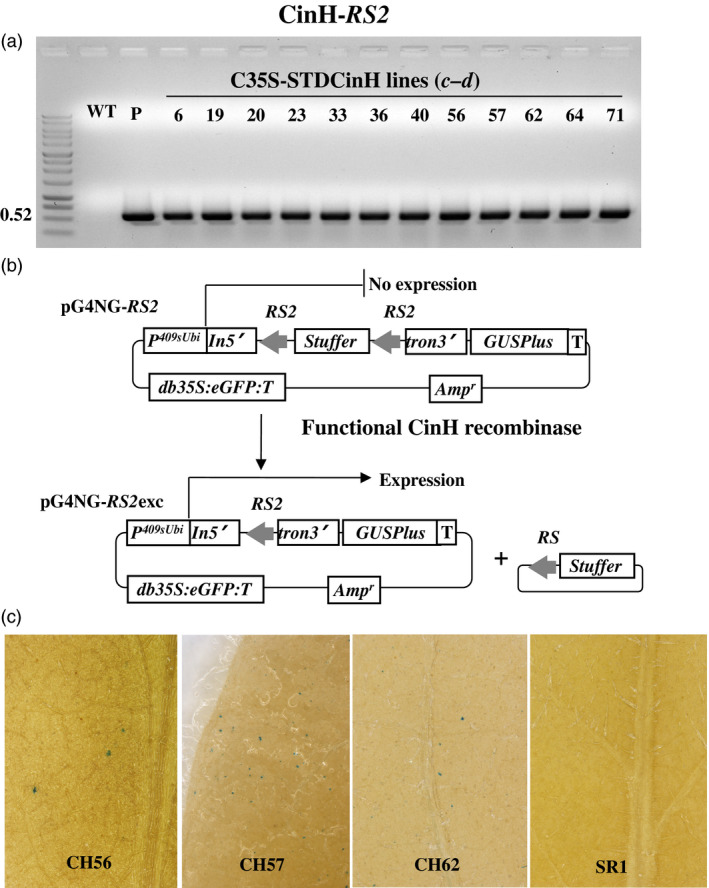
CinH transgenic plants and expression activity detection. (a). PCR analysis of kanamycin‐resistant regenerants. DNA from kanamycin‐resistant shoots was amplified by primer pair ***c–d*** (Figure 1b, Table 1) to give a 0.68 or 0.52 fragment that includes part of the 35S promoter, stroma targeting domain and part of the *CinH* coding region. Amplified DNA products are from WT—untransformed tobacco negative control, P—C35SSTDCinH as positive controls and kanamycin resistant shoots (numbered lanes). (b). Schematic representative of constructions and strategy to detect recombinase activity in a transient assay. In vector pG4NG‐*RS2*, GUSPlus reporter gene expression is inhibited due to the presence of a terminator‐rich ‘stuffer’ sequence in the intron (In5'tron3’). This stuffer will be excised by site‐specific recombination in cells containing CinH. The resultant pG4NG‐*RS2*exc plasmid expresses the GUSPlus reporter. *p409sUbi*—potato ubiquitin promoter; *T*—3'Nos terminator; *In5’*—maize ubiquitin first intron 5’ fragment; *tron3’*—maize ubiquitin first intron 3’ fragment; *GUSPlus*—β‐glucuronidase gene sequence; *db35S*—double enhanced CaMV 35S promoter; *eGFP*—enhanced Green Fluorescent Protein gene; *RS2*—CinH recombinase recognition sequences; Amp—Ampicillin resistance gene. (c). Detection of site‐specific recombinase activity with the pG4NG‐*RS2* vector. Leaves from C35SSTDCinH transgenic plants after bombardment with the pG4NG‐*RS2* plasmid, histochemical staining to detect β‐glucuronidase activity, (blue spots) indicating recombinase‐mediated excision and gene activation. Leaves were from WT, nontransformed SR1 tobacco, the negative control, three *CinH* expression transgenic plants (CH56, CH57 and CH62).

Utilizing Agrobacterium‐mediated transformation, we obtained at least 50 kanamycin resistant SR1 tobacco plants each that contained pC35‐STDParAo or C35‐STDCinH T0 (Figure 1b). PCR analyses using primer pair ***c–d*** (Figure 1b, Table 1) confirmed the presence of the parAo or cinH transgenes (Figures 3a, 4a) in 19 of 55 ParA and 12 of 72 CinH resistant primary transformants. To confirm that the recombinases were functionally active in the transgenic tobacco plants that contained the PCR product, we employed a recombinase activity‐histochemical staining transient assay we previously developed (Blechl *et al*., 2012; Shao *et al*., 2014). Plasmids designed to detect ParA or CinH activity (pG4NG‐MRS or pG4NG‐RS2; Figures 3b, 4b, respectively) were bombarded into tobacco leaves. The GUSPlus reporter gene in pG4NG‐MRS and pG4NG‐RS2 is not expressed due to the presence of a transcription terminating stuffer sequence in the St409Ubi promoter first intron. When the activity detection vectors were introduced into cells containing their corresponding active‐specific recombinases, the res‐flanked stuffer will be excised (Figures 3b, 4b), and the GUSPlus gene expression can then be detected by histochemical staining (Figures 3c, 4c). This assay was used to screen 19 T0 ParAo and 12 T0 CinH lines. A total of 10 of 19 (ParA) and 9 of 12 (CinH) plant lines exhibited detectable levels of recombinase‐mediated recombination manifested in β‐glucuronidase activity; three ParA and three CinH active lines are shown in Figures 3c and 4c. For each recombinase, we chose three primary transgenic plants (ParA—PAo4, PAo6 and PAo18; CinH—CH56, CH57 and CH62) that produced T1 progeny that segregated 3:1 for kanamycin resistance and exhibited consistently high levels of recombinase activity in the transient assay. These T0 plants were used as pollen donors in crosses to test ParA and CinH excision from the plastid genome.

### Excision by ParA‐*MRS* and CinH‐*RS2* systems

**Figure 5:**
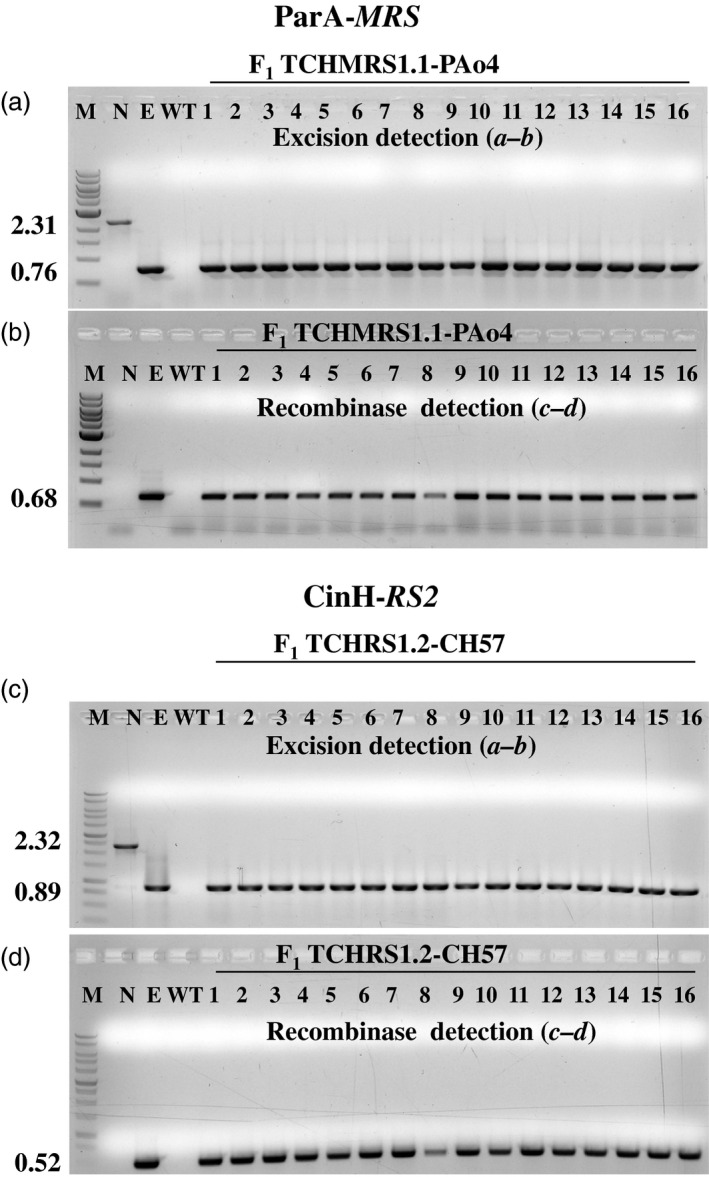
PCR analysis to detect site‐specific excision and recombinase encoding genes in F_1_ progeny. PCR analysis with primer pair ***a–b*** (upper panels a, c) and ***c‐d*** (lower panels b, d) on 16 randomly kanamycin and spectinomycin selected F_1_ hybrid plants from crosses of THCMRS1.1 by PAo4 (panels a, b) and TCHRS1.2 by CH57 (c, d). Amplified products are from DNA from WT—nontransformed tobacco SR1, negative control; N—pTCH‐*MRS* or pTCH‐*RS2* plasmids*;* E—pTCH‐*MRS* exc or pTCH‐*RS2*exc, plasmids after excision and F_1_ progeny of the crosses (numbered lanes). The sizes of the amplicons are indicated to the left. M: DNA size markers.

**Figure 6:**
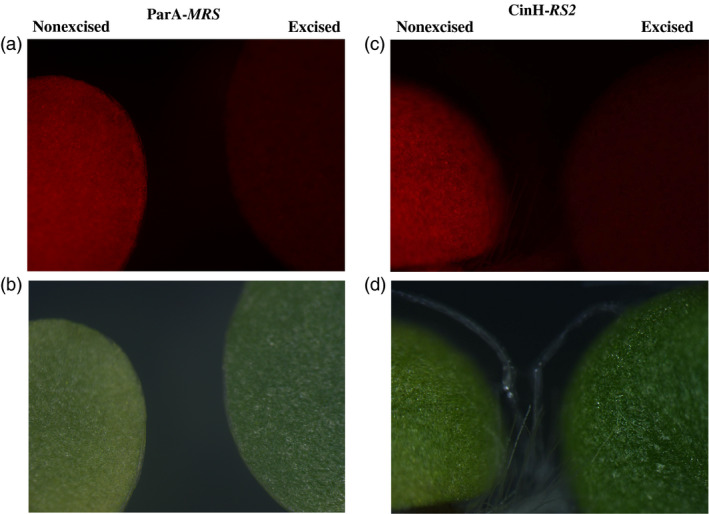
*DsRed* expression in leaves of pTCH‐*MRS* and pTCH‐*RS2* transformed plants and their F_1_ progeny under green light excitation. (a). Fluorescence microscopic images of leaves from a TCH‐*MRS* transformed plant (nonexcised) and its F_1_ progeny from a cross with pollen from a ParAo recombinase expression plant (excised). (b). Bright‐field (lower) microscopic images of leaves from a TCH‐*MRS* transformed plant (nonexcised) and its F_1_ progeny from a cross with pollen from a ParAo recombinase expression plant (excised). (c). Fluorescence microscopic images of leaves from a TCH‐*RS2* transformed plant (nonexcised) and its F_1_ progeny from a cross with pollen from a CinHwt recombinase expression plant (excised). (d). Bright‐field (lower) microscopic images of leaves from a TCH‐*RS2* transformed plant (nonexcised) and its F_1_ progeny from a cross with pollen from a CinHwt recombinase expression plant (excised).

To generate ParA‐ and CinH‐mediated marker gene excision events, the T_0_ TCH‐*MRS* and TCH‐*RS2* target plants were used as females in crosses with pollen from each of the three C35‐STDParAo plants (PAo4, PAo6 and PAo18) and C35‐STDCinH plants (CH56, CH57 and CH62). F_1_ seeds from each of the eighteen crosses were germinated on MS medium with 100 μg/mL kanamycin and 500 mg/L spectinomycin. DNA from plants surviving the dual selection was assayed by PCR for the presence of the target locus with primers ***a–b*** (Figure 1a) and the presence of the recombinase *parAo* and *cinH* genes with primers ***c–d*** (Figure 1b). For each cross, twenty of twenty F_1_ plants contained the recombinase gene, as evidenced by a *c‐d* amplicon of 0.68 kbp for the PAo4 crosses and 0.52 kbp for the CinH crosses. Typical results for 16 plants from one cross of each are shown in Figure 5a and c, respectively. Primer pair ***a–b*** detected only the excision product of 0.76 kb (ParA‐*MRS*) or 0.89 kb (CinH‐*RS2*) in DNAs from these same plants (Figure 5a and c, respectively). There is no trace of the 2.31 kb or 2.32 kb ***a–b*** amplicons expected for unexcised TCH‐*MRS* (Lane ‘N’ in Figure 5a) and TCH‐*RS2* (Lane ‘N’ in Figure 5c) target DNAs, respectively. Evidence of complete excision was obtained in all F_1_ progeny of each successful cross. These results are similar to the successful excision observed in Cre‐lox (Corneille *et al*., 2001) and Bxb1 systems (Shao *et al*., 2014) and could be classified as a strong activator owing to the observed 100% excision in the seedling tested. We sequenced the 0.76‐kb (ParA‐*MRS*) and 0.89‐kb (CinH‐*RS2*) PCR amplicons from the F_1_ DNA of six independent crosses and found perfectly conserved *MRS* or *RS2* sites in each one (Figure 1d, e). This confirmed that the ParA‐ or CinH‐mediated excision was site specific. Seeking further evidence for the completeness of excision, we examined the leaves from F_1_ hybrid plants under green light (550/25 excitation) with a fluorescent light microscope. No DsRed fluorescence was detected (Figure 6, excised), indicating an absence of a functional *DsRed* gene. In contrast, the maternal parents transplastomic for TCH‐*MRS* or TCH‐*RS2* exhibit red fluorescence under these conditions (Figure 6, nonexcised). Otherwise, the F_1_ hybrids are similar in phenotype to their maternal parents.

### Analysis of F_2_ progeny

**Figure 7:**
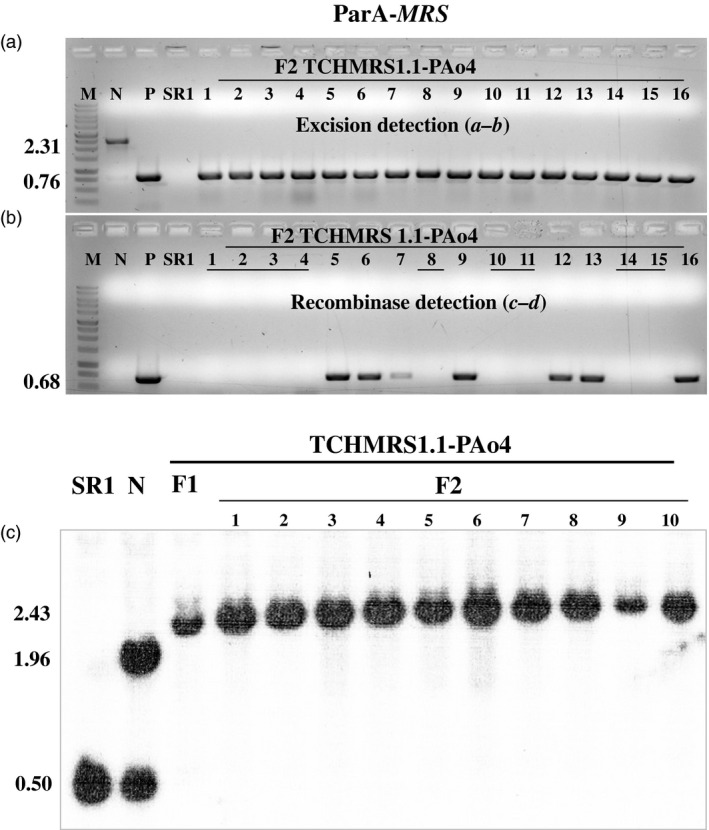
Molecular analysis for segregation of ParA site‐specific excised transplastomic DNA from the presence of the recombinase expression gene in F_2_ tobacco plants. (a). PCR products of DNAs from F_2_ progeny (numbered lanes) of the TCHMRS1.1‐PAo4 cross amplified with primers ***a–b***. Determines the presence of excised (0.76 kb) or unexcised (2.31 kb) target. (b). PCR products of DNAs from F_2_ progeny (numbered lanes) of the TCHMRS1.1‐PAo4 cross amplified with primers ***c–d***. Amplicon (0.68 kb) determines presence of *parAo* gene. The underlined numbers in panel b are F_2_ plants that contain the site‐specific excision product (panal a), but not the recombinase gene. Amplified products are from DNA from WT—nontransformed tobacco SR1, negative control; N—pTCH‐*MRS* plasmid*;* E—pTCH‐*MRS* exc, plasmid after excision. The sizes of the amplicons are shown to the left. M: DNA size markers. (c). Southern blot analysis of transplastomic plants with probe TRN (grey bar below diagram in Figure 1a). DNAs from untransformed (WT; 0.50 bp) and transplastomic TCHMRS1.1 plants before (N lanes; 1.96 kb) and after crossing to PAo4 (F_1_ lane; 2.43 kb) and the F_2_ progeny of the crosses (numbered lanes) were digested with *Bam* HI and *Bgl* II. The sizes in kb of the hybridizing fragments are shown to the left.

**Figure 8:**
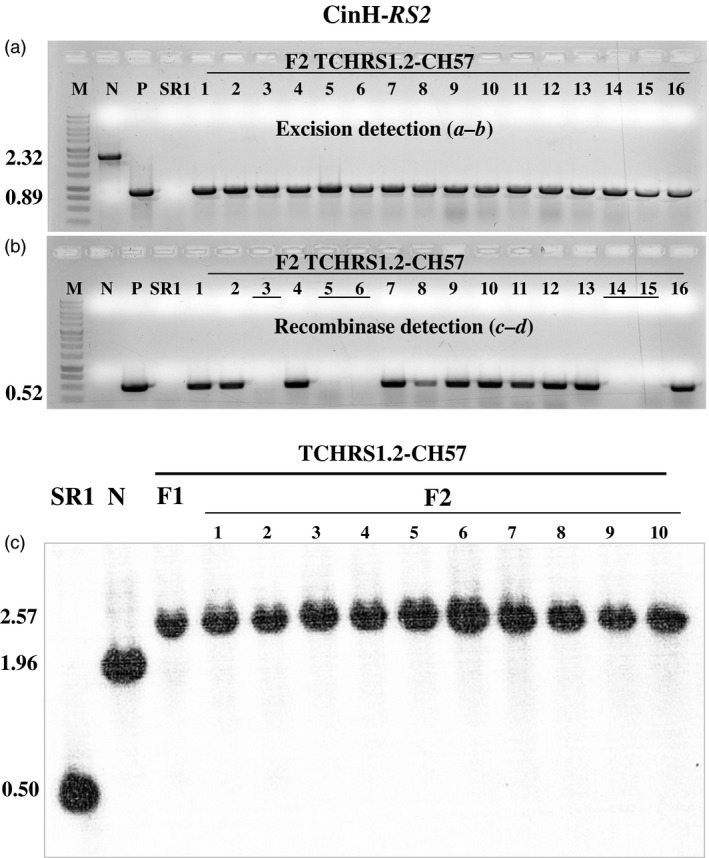
Molecular analysis for segregation of CinH site‐specific excised transplastomic DNA from the presence of the recombinase expression gene in F_2_ tobacco plants. (a). PCR products of DNAs from F_2_ progeny (numbered lanes) of the TCHRS1.2‐CH57 cross amplified with primers ***a–b*** (panel a). Determines the presence of excised (0.89 kb) or unexcised (2.32 kb) target. (b). PCR products of DNAs from F_2_ progeny (numbered lanes) of the TCHRS1.2‐CH57 cross amplified with primers ***c–d***. Amplicon (0.52 kb) determine the presence of *cinH* gene. The underlined numbers in panel b are F_2_ plants that contain the site‐specific excision product (panal a), but not the recombinase gene. Amplified products from panel a are from DNA from WT—nontransformed tobacco SR1, negative control; N—pTCH‐*RS2* plasmid*;* E—pTCH‐*RS2*exc, plasmid after excision. The sizes of the amplicons are shown to the left. M: DNA size markers. (c). Southern blot analysis of transplastomic plants with probe TRN (grey bar below diagram in Fig. 1a). DNAs from untransformed (WT; 0.5 kb) and transplastomic TCHRS1.2 plants before (N lanes; 1.96 kb) and after crossing to CH57 (F_1_ lane; 2.57 kb) and the F_2_ progeny of the cross (numbered lanes) were digested with *Bam* HI and *Bgl* II. The sizes in kb of the hybridizing fragments are shown to the left.

F_1_ positive for excision plants (discussed above) was allowed to self‐fertilize and set seeds. F_2_ plants were grown in the absence of selection to allow segregation ratios to be estimated. DNA from the F_2_ progeny was tested by PCR for the presence or absence of the excision and recombinase amplicons using primer pairs ***a–b*** and ***c–d***, respectively (Figures 7 and 8, Table 2). As expected for maternal inheritance, all F_2_ plants contained the band from the target DNA (Figures 7a and 8a). Moreover, in all cases, the only amplicon detected was that from the target DNA after excision (0.89 or 0.76 kb, Figure 1c). Most but not all (Table 2) of the F_2_ progeny contained the recombinase (e.g. lanes 5–7, 9, 12–13 and 16 in Figure 7b and lanes 1, 2, 4, 7–13 and 16 Figure 8b). This is consistent with nuclear gene inheritance, although some of the families did not appear to exhibit 3:1 segregation (Table 2). The presence of excised plastid target in the absence of the recombinase‐coding genes indicates that excision products of ParA‐*MRS* and CinH‐*RS2* systems are transmitted to progeny via plastid inheritance. To further investigate the lines for complete loss of the *DSRed* gene after excision, we used PCR to test for its presence in the F_2_ progeny. Nine progeny plants were randomly chosen from both ParA‐ and CinH‐mediated excision events (Figure S1). The *DSRed* gene amplicon was not detected in the F_2_ progeny analysed although its presence was observed in the original unexcised target lines. These results indicate that the excised DNA was not reintegrated into the genome.

**Table 2:** PCR analysis of F_2_ TCH‐*MRS* and TCH‐*RS2* plants

	TargetParent	Recombinase Parent	F2 Plants tested	Positive for target locus^a^	Positive for recombinase gene^b^	Positive for excision and negative for recombinase gene^c^
ParA‐*MRS*	TCH‐MRS1.1	PAo4	20	20	14	6
	TCH‐MRS1.1	PAo6	20	20	14	6
	TCH‐MRS1.1	PAo18	20	20	15	5
	TCH‐MRS2.1	PAo4	20	20	9^e^	11
	TCH‐MRS2.1	PAo6	20	20	10^e^	10
	TCH‐MSR2.1	PAo18	20	20	12	8
	TCH‐MRS3.1	PAo4	20	20	6^e^	14
	TCH‐MRS3.1	PAo6	20	20	13	7
	TCH‐MRS3.1	PAo18	20	20	13	7
CinH‐*RS2*	TCH‐RS21.1	CH56	20	20	13	7
	TCH‐RS21.1	CH57	20	20	15	5
	TCH‐RS21.1	CH62	20	20	15	5
	TCH‐RS22.1	CH56	20	20	15	5
	TCH‐RS22.1	CH57	20	20	14	6
	TCH‐RS22.1	CH62	20	20	17	3
	TCH‐RS23.1	CH56	20	20	12	8
	TCH‐RS23.1	CH57	20	20	12	8
	TCH‐RS23.1	CH62	20	20	11^d^	9

a Primers ***a*** and ***b*** yielded the 2.31 and/or 0.76 kb (ParA‐*MRS*), or 2.32 and/or 0.89 kb (CinH‐*RS2*) target fragment.

b Primers ***c*** and ***d*** yielded the 0.68 kb *ParAo* fragment, or 0.52 kb *CinHwt* gene.

c Primers ***a*** and ***b*** yielded the 0.76 kb (ParA‐*MRS*) or 0.89 kb (CinH‐*RS2*) excision fragment and failed to detect a 2.31 kb (ParA‐*MRS*) or 2.32 kb (CinH‐*RS2*) target fragment, and primers ***c*** and ***d*** failed to detect the 0.68 kb *ParAo* or 0.52 kb *CinHwt* gene.

d Fit to 3:1 segregation ratio has < 0.05 by chi‐squared test.

e Fit to 3:1 segregation ratio has <0.01 by chi‐squared test.

Southern blots were performed to further characterize the ParA‐ and CinH‐mediated excision events in DNA from F_1_ and F_2_ plants and their transgenic parents. DNA from these plants was digested with *Bam*HI and *Bgl*II and hybridized with a ^32^P‐labelled probe amplified with primers from the *trnI* gene (Table 1). In parental plants, the 1.96‐kb fragment characteristic of the intact, nonrecombined target ParA‐*MRS* and CinH‐*RS2* DNAs (Lanes N in Figures 7c and 8c) along with the 0.5 kb intact wild‐type plastid *trnI* gene (Lanes ‘SR1’ and N in Figures 7c and 8c) are found. In the F_1_ hybrids, both the 0.5 and 1.96‐kb band are absent; only a 2.43‐kb or 2.57‐kb bands representing the excision product was detected in all lines containing a recombinase gene *parAo* or *CinH*, respectively. Furthermore, the all tested F_2_ progeny of the hybrid lines exhibited the expected band of 2.43‐kb or 2.57‐kb recombined product, while the recombinase genes were absent 30%–40% of the time suggesting that ParA and CinH transplastomic excision had been transmitted to next generation in the absence of the recombinase gene.

## Conclusion

Plant biotechnology is widely used to improve agronomically important crop plants. In recent years, transplastomic plants have become an improved method of genetic engineering for commercialization of important traits. To date, 114 transgenes have been used to modify a variety of plant chloroplasts with uses ranging from stress tolerance to synthesis of biomaterials to enhancing nutrition and health products (Daniell *et al*., 2016). However, all transgene modifications rely on the introduction of a selectable marker gene due to the low efficiency of the initial transgene integration. Selectable maker genes become unwanted DNA once homoplastic transgenic plants are obtained, and because they are a perceived risk for wide‐scale deployment from transgenic plants within the environment. Gene stacking or the addition of more genes after an initial successful transgenic plant is produce requires the use of another selection marker gene if the original is not removed. Unfortunately, there is a limited number of selection genes available for transplastomic plant production. In fact, only the gene conferring spectinomycin resistance is predominantly used. Therefore, without an efficient marker removal system, further gene stacking would be hampered. A number of reports for the effective removal of unwanted DNA from transgenic plant genomes by site‐specific recombination systems have been published (Ballester *et al*., 2006; Blechl *et al*., 2012; Cao *et al*., 2006; Cuellar *et al*., 2006; Djukanovic *et al*., 2008; Hu *et al*., 2008; Kempe *et al*., 2010; Luo *et al*., 2007; Mlynarova *et al*., 2006; Nanto and Ebinuma, 2008; Nanto *et al*., 2009; Thomson *et al*., 2009, 2010). To date, the Cre/*loxP*, phiC31/*att* and Bxb1/*att* site‐specific recombination systems have been shown to successfully excise DNA from plastid genomes (Corneille *et al*., 2001; Kittiwongwattana *et al*., 2007; Lutz *et al*., 2006; Shao *et al*., 2014). Cre/*loxP* is a bidirectional system, where the enzyme Cre recognizes two identical loxP sites. While this is an exceptionally active recombinase system, the presence of identical loxP sites appears to lead to instability when used in the plastid genome (Corneille *et al*., 2001; Hajdukiewicz *et al*., 2001). Thus, large serine recombinase systems were considered as alternative options for marker excision. The large serine recombinase systems recognize two unique recognition sites *attP* and *attB*, and unidirectionally perform recombination to yield the hybrid product sites known as *attL* and *attR* (Wang *et al*., 2011). The *attL* and *attR* sites are no longer recognized by the recombinase enzyme in the absence of an additional excisionase protein. Further, the nonhomologous nature of the Bxb1 and PhiC31 recognition sites appear to offer reliable activity and greater stability when used in the plastid genome (Kittiwongwattana *et al*., 2007; Shao *et al*., 2014). To increase the tools available for plastid selection marker removal, we investigated the potential of the small serine recombinases CinH and ParA for excision in the chloroplast. Results clearly demonstrate that the CinH/*RS2* and ParA/*MRS* systems are efficient at performing plastid genome excision and did not exhibit the instability seen by the Cre/*loxP* system. Complete excision (100%) was observed for every line tested, demonstrating that the CinH/*RS2* and ParA/*MRS* systems provide a very reliable means of plastid marker removal. While beyond the scope of this study and based on empirical observations only the small serine recombinases appear more reliable than the large serine for chloroplast‐mediated excision. This may be due to the difference in protein size and rate of import into the chloroplast. However, these observations do not detract from the utility of the large serine recombinases for chloroplast manipulation. In fact now that a series of recombinase systems have been shown to function in the plastid, some of which can mediate stable integration (large serine recombinase), it may be possible to produce chloroplast ‘founder’ lines that enable precise sequential gene stacking, without the need to make vectors for homologous recombination for each unique targeting event. The large serine recombinase can be utilized to target the genome in a precise manner (Lutz *et al*., 2004) bringing in the DNA of choice and include the selection marker. With correct insertion confirmed, the small serine recombinase can be employed to remove unneeded DNA such as the selectable marker and or backbone DNA of an integrating plasmid in a site‐specific manner that allows recycling the system. If designed properly, this technique can be reused indefinitely for sequential gene stacking with preassembled plasmids (Wang *et al*., 2011).

## Methods

### Construction of the target and recombinase vectors

The tobacco plastid transformation vectors, pTCH‐*MRS* and pTCH‐*RS2* (GeneBank accession numbers KY426959 and KY426960, respectively), were designed to test the recombination capabilities of the ParA and CinH systems, respectively. *Not*I‐*MRS*‐DsRed‐*MRS*‐*Not*I and *Not*I‐*RS2*‐DsRed‐*RS2*‐*Not*I synthesized fragments were inserted at the *Not*I sites in the tobacco plastid transformation vector (Guda *et al*., 2000) as described (Shao *et al*., 2014). Figure 1a shows the schematic of the vectors before ParA‐ or CinH‐mediated excision and Figure 1c after ParA or CinH‐mediated excision. The pTCH‐MRSexc or pTCH‐RS2exc control vectors amplified as excision controls (‘E’ lanes in Figures 5, 7, 8) were generated by removal of the *DsRed* region by ParA or CinH recombinase‐mediated excision in *E. coli*.

The recombinase expression vectors for nuclear DNA transformation, pC35‐STDParAo and pC35‐STDCinH (Figure 1b; (GeneBank accession numbers KY426961 and KY426962, respectively) contain coding regions for a plant codon‐optimized serine resolvase ParA (named ParAo), and a wild‐type serine recombinase CinH, respectively. Each coding sequence is fused to the stroma‐targeting domain from ribulose 1, 5‐bisphosphate carboxylase small subunit gene of tobacco (STD). The STDParAo and STDCinH open reading frames were synthesized (Genewiz, South Plainfield, NJ) with 5’*Asc*I and 3’*Spe*I sites and inserted into p35S‐ParA (Thomson *et al*., 2009) cut with *Asc*I and *Spe*I to generate the expression cassettes p35S‐STDParAo and p35S‐STDCinH. After confirmation of its DNA sequence, the expression cassettes of p35S‐STDParAo and p35S‐STDCinH were excised from the pUC backbone using the *Hin*dIII and *Sac*I restriction sites and inserted into the pCAMBIA2300 (http://www.cambia.org/daisy/cambia/home.html), a binary vector with the *npt*II (neomycin phosphotransferase II gene) SMG.

### Nuclear transformation of target vectors and testing recombination activity

*Agrobacterium tumefaciens* AGLI was used for leaf disc transformation of tobacco (*Nicotiana tabacum* cv. Petit Havana SR1) (Horsch *et al*., 1985; Maliga *et al*., 1973). Primary tobacco transformants were selected on MS medium (Sigma, St. Louis, MO), with 3% sucrose, 0.5% phytoblend (Cassion Labs, Smithfield, UT) 3 mg/L 6‐Benzylaminopurine (6‐BA) and 100 mg/L kanamycin. Rooted plantlets were obtained after 6 weeks that can be transferred to soil. PCR analysis was carried out to confirm that the shoots were transgenic.

To detect recombinase activity in transgenic plants, pG4NG‐*MRS* and pG4NG‐*RS2* (Figures 3a and 4a) detection vectors were constructed. Each detection vector includes an ParA or CinH *res*‐flanked transcription terminator cassette embedded as a stuffer fragment within the potato St409Ubi promoter first intron (Rockhold *et al*., 2008). The presence of this terminator cassette blocks St409Ubi promoter‐mediated expression of the *Staphylococcus* sp. *GUSPlus* reporter gene using a modification of the method described by Blechl *et al*., 2012;. ParA‐ or CinH‐mediated recombination is expected to remove the *res*‐flanked terminator cassette from the intron, allowing uninterrupted *GUSPlus* transcription from the St409Ubi promoter (Figures 3a and 4a) and detection of β‐glucuronidase (GUS) enzyme activity, as described below.

Detection of recombinase activity in plants transformed with pC35‐STDParAo or pC35‐STDCinH was carried out as previously described (Shao *et al*., 2014). Briefly, the T_0_ leaf tissue was arranged to cover at least 2.5 cm in the centre of the Petri plate containing sterile filter paper and was bombarded with 1.0 μm gold particles (Seashell Inc., La Jolla, CA) coated as specified by the manufacturer with a total of 1.0 mg of pG4NG‐*MRS* or pG4NG‐*RS2* plasmid DNA. After 16 h, the tissues were histochemically stained for GUS activity with 1 mm X‐Gluc (Gold Biotechnologies, St. Louis, MO) as previously described (Jefferson *et al*., 1987). After staining, the leaves were treated with 70% ethanol to remove the chlorophyll. Transformed plants exhibiting a high level of ParA or CinH activity, based on the density of the blue spots in GUS staining, were identified (Figures 3c and 4c) and used in crosses with the transplastomic tobacco plants carrying pTCH‐*MRS* or pTCH‐*RS2*.

For a list of vectors created and functional applications of each, see Table 3.

**Table 3:** Vectors and function

Vector Name	Gene Bank Assession	Purpose	Type
pTCH‐*MRS*	KY426959	Tobacco chloroplast targeting vector for ParA excision testing. DSRed flanked by MRS sites	Plastid
pTCH‐*RS2*	KY426960	Tobacco chloroplast targeting vector for CinH excision testing. DSRed flanked by RS2 sites	Plastid
pG4NG‐*MRS*	ND	ParA recombinase activity assay vector. Used to determine whether a recombinase expressing plant line is active. Beta galactosidase is activated in the presence of functional ParA	Nuclear Transient assay
pG4NG‐*RS2*	ND	CinH recombinase activity assay vector. Used to determine whether a recombinase expressing plant line is active. Beta galactosidase is activated in the presence of functional CinH	Nuclear Transient assay
pC35‐STDParAo	KY426961	Codon optimized stroma targeted ParA gene constitutively expressed by 35S promoter. Lines generated and crossed with TCH‐*MRS* lines to test ParA activity in chloroplast genome	Nuclear
pC35‐STDCinH	KY426962	Stroma targeted CinH gene constitutively expressed by 35S promoter. Lines generated and crossed with TCH‐*RS2* lines to test CinH activity in chloroplast genome	Nuclear

### Plastome transformation and plant regeneration

Plastomic transformation was carried out by the biolistic protocol, as previously described (Lutz *et al*., 2006; Verma *et al*., 2008). Briefly, leaves of tobacco (*Nicotiana tabacum* cv. Petit Havana SR1) grown in sterile culture to the 5‐7 leaf stage were placed abaxial side up on filter paper, and transforming DNA was introduced by the biolistic DNA delivery system using 0.6 μm gold particles (Bio‐Red, Hercules, CA), 1100 psi rupture disks, the target plate holder on the forth shelf from top (9 cm below the microcarrier launch assembly). After 2 days in the dark, the leaves were cut into 5 mm^2^ pieces and placed on RMOP (MS salts (Caisson), 100 mg/L myo‐inositol, 1 mg/L thiamine HCl, 1 mg/L BAP, 0.1 mg/L NAA, 30 g/L sucrose, pH 5.8, 6 g/L phytoblend, 500 mg/L spectinomycin) media for the first round of selection. After 4–8 weeks in the same plates, the spectinomycin resistant clones appear. Total DNA from the putative transplastomic shoots was screened for the presence of the transgene by PCR with the primers of ***e–f***. Leaves from PCR‐positive plants were cut into 2 mm^2^ pieces and placed on RMOP selection medium for the second round of selection. After 3–4 weeks, the regenerated shoots were transferred to rooting medium containing 500 mg/L spectinomycin. Total DNA was isolated from the regenerated plant leaves characterized by PCR and Southern blot as described below.

### PCR analysis

PCR analyses were performed to confirm that putative transgenic and transplastomic plants contained transgenes and that excision had occurred after crosses between the two types of plants. Genomic and plastid DNA was extracted as described (Shao *et al*., 2014). A single leaf piece (25‐mm^2^) was ground in 400 mL of buffer (200 mm Tris‐HCl pH 7.8, 250 mm NaCl, 25 mm EDTA, 0.5% SDS). After centrifugation and isopropanol precipitation, the pellet was washed with 70% ethanol and resuspended in 50 μL of water. PCR amplification was performed using 2 μL of genomic DNA in reactions with a total volume of 25 μL. The primers and conditions used for PCR analysis are listed in Table 1; their locations in the vectors are depicted in Figure 1a–c. Products were separated by electrophoresis in 0.8% (w/v) agarose. Gel images were digitized with a resolution of 200 dpi in a black on white background TIF format.

### Southern blot analysis of DNA

For Southern blot analysis, genomic DNA was extracted from aerial tissues of transgenic and wild‐type control plants using a modification of the method described by Dellaporta *et al*., 1983;. DNAs of control and transgenic plants were digested with *Bgl*II and *Bam*H1 for 6 h at 37 °C and separated by electrophoresis on a 0.8% (w/v) agarose gel. The DNA was then transferred to a Hybond‐N membrane (Amersham) and hybridized with the ^32^P‐labelled probe 0.50 kb TRN (Gray bar in Figure 1a) using Taq^TM^ polymerase (Promega, Madison, WI) with the primers TRN1.2 F63 and TRN1.2 R63 (Table 1).

### Crossing of transgenic plants

Each transplastomic T_0_ plant (*MRS* or *RS2*) was crossed with each of three plant lines exhibiting high levels of ParA or CinH activity, respectively. A total of eighteen independent crosses (nine for ParA‐*MRS* system, nine for CinH‐*RS2* system) of T_0_ transplastomic × T_0_ transgenic pollen were made. Seeds that germinated on MS media containing 500 mg/L spectinomycin and 100 mg/L kanamycin were transferred into greenhouse for growth. Nontransgenic tobacco seeds did not survive on spectinomycin and kanamycin selection media.

### Progeny analysis

F_2_ seeds were produced by self‐pollination of F_1_ plants that had been shown to carry recombinase‐mediated excisions of the *DsRed* marker gene. The F_2_ seeds were germinated on MS medium without any selection. After 10–12 days, total plant DNA was extracted from the aerial tissues of the seedlings and subjected to PCR analyses, using primers ***a–b*** to show excision within the target transgene and ***c–d*** to detect the presence or absence of the recombinase ORF.

### Fluorescence microscopy

F_1_ seeds produced from crosses of T_0_ recombinase pollen with transplastomic target lines were germinated on MS medium without selection. After 14 days, detection of *DsRed* expression was performed by fluorescence microscopy (Figure 6) with an AxioImager Z1 microscope (Carl Zeiss, Gottingen, Germany) and filter set 43HE. Images were captured using an AxioCam MRm camera and AxioVision Release 4.2 software (Carl Zeiss).

## Competing interests

The authors declare that they have no competing interests.

## Authors’ contributions

JT designed the experiments. MS performed the experiments. JT, MS and AB performed the data analysis. The article was written by JT and AB. All authors read and approved the final manuscript.

## Supporting information

Supplementary File
