# Supplementary material for: Small serine recombination systems ParA‐ MRS and CinH‐ RS2 perform precise excision of plastid DNA
Source: Plant Biotechnol J. 2017 May 16;15(12):1577–89. doi: 10.1111/pbi.12740 (PMC5698047; doi:10.1111/pbi.12740)
Supplement: Supplementary file 1 — Supplementary File [file PBI-15-1577-s001.pdf]

Shao et al. Supplemental
Figure S1

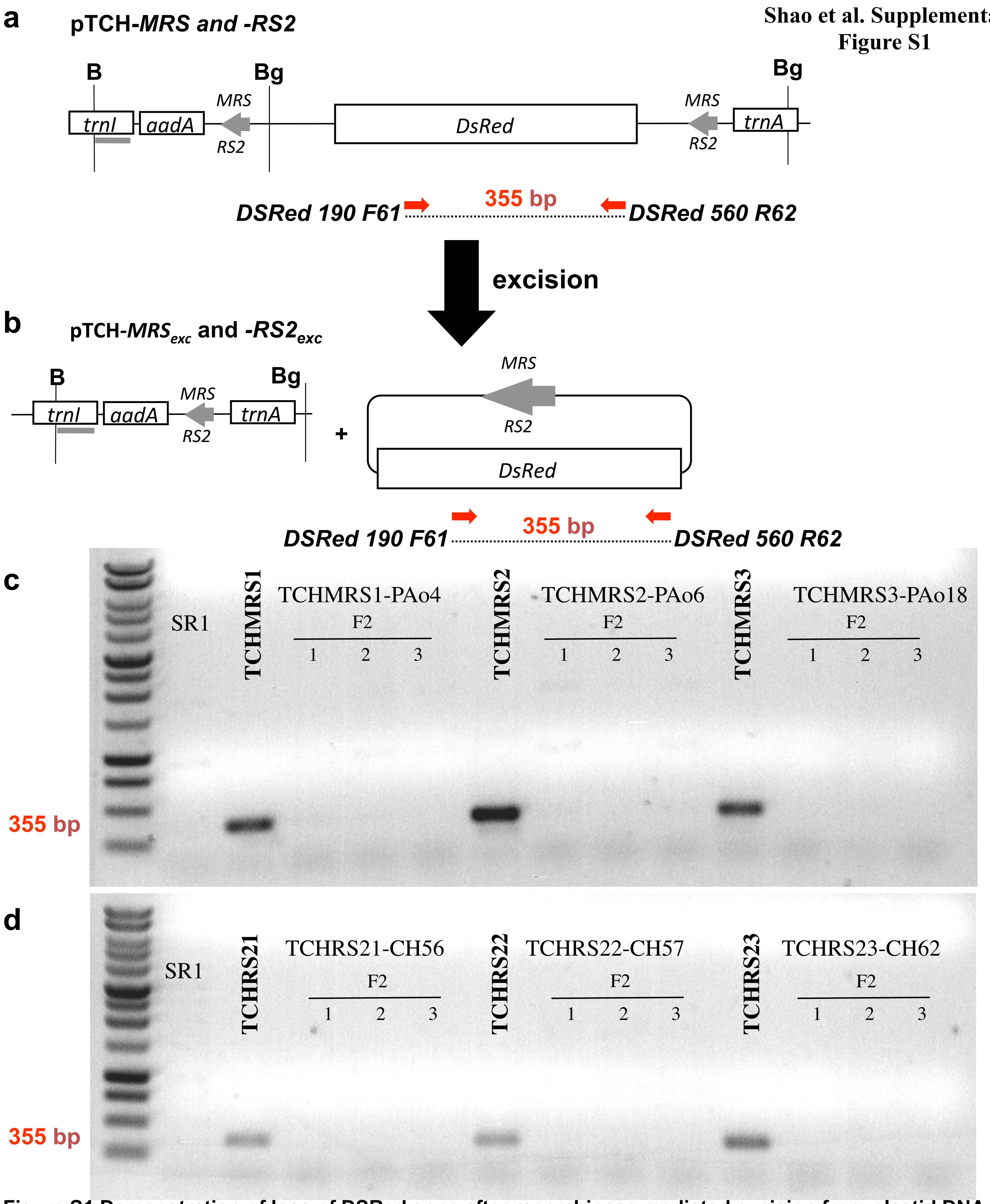

Figure S1 Demonstration of loss of DSRed gene after recombinase mediated excision from plastid DNA by ParA-*MRS* and CinH-*RS2* systems in F_2 progeny.
Panels **a** and **b** schematic from Figure 1. with *DSRed* gene expanded to show placement of *DSRed 190 F60* and *DSRed 560 R62* primers. Panels **c** and **d** Amplified products are from DNA of SR1 – non-transformed tobacco, negative control; TCH-*MRS* or TCH-*RS2* transplastomic DNA, positive control; F_2 progeny of the crosses described in Table 2 (numbered lanes). The sizes of the amplicons are indicated to the left. Primer sequences Table 1.
